# miR-381-3p knockdown improves intestinal epithelial proliferation and barrier function after intestinal ischemia/reperfusion injury by targeting nurr1

**DOI:** 10.1038/s41419-018-0450-z

**Published:** 2018-03-14

**Authors:** Liwei Liu, Jihong Yao, Zhenlu Li, Guo Zu, Dongcheng Feng, Yang Li, Wasim Qasim, Su Zhang, Tong Li, Huizhi Zeng, Xiaofeng Tian

**Affiliations:** 1 Department of General Surgery, The Second Affiliated Hospital of Dalian Medical University, Dalian, 116023 China; 2 Department of Pharmacology, Dalian Medical University, Dalian, 116044 China

## Abstract

Impairment in gut barrier function induced by intestinal ischemia/reperfusion (I/R) injury is associated with high morbidity and mortality. Intestinal barrier function requires the tight coordination of epithelial migration, proliferation and differentiation. We previously observed that nuclear receptor-related protein 1 (nurr1)-mediated proliferative pathway was impaired in intestinal I/R injury. Here, we aimed to assess the effect of nurr1 on intestinal barrier function and to evaluate microRNA (miRNA)-nurr1-mediated restoration of intestinal barrier function in intestinal I/R injury. We induced an in vivo intestinal I/R injury mouse model by clamping and then releasing the superior mesenteric artery. We also performed an in vitro study in which we exposed Caco-2 and IEC-6 cells to hypoxia/reoxygenation (H/R) conditions to stimulate intestinal I/R injury. Our results demonstrated that nurr1 regulated intestinal epithelial development and barrier function after intestinal I/R injury. miR-381-3p, which directly suppressed nurr1 translation, was identified by microarray and bioinformatics analysis. miR-381-3p inhibition enhanced intestinal epithelial proliferation and barrier function in vitro and in vivo and also attenuated remote organ injury and improved survival. Importantly, nurr1 played an indispensable role in the protective effect of miR-381-3p inhibition. Collectively, these findings show that miR-381-3p inhibition mitigates intestinal I/R injury by enhancing nurr1-mediated intestinal epithelial proliferation and barrier function. This discovery may lead to the development of therapeutic interventions for intestinal I/R injury.

## Introduction

The damage sustained by ischemic intestinal tissue as a result of the activation of vicious cascades during the restoration of blood flow is known as intestinal ischemia/reperfusion (I/R) injury. Intestinal I/R injury is a common life-threatening complication observed in many clinical conditions, such as mesenteric arterial thrombosis, small bowel volvulus, abdominal aortic aneurysm surgery, hemorrhagic shock and sepsis^1–3^. Intestinal ischemia causes severe cellular damage that provokes epithelial barrier dysfunction during reperfusion. Intestinal epithelial barrier loss leads to increases in permeability and bacterial translocation^4,5^. The resulting intestinal barrier dysfunction is a key factor in the aggravation of the deleterious complications of intestinal I/R, including systemic inflammatory response syndrome and multiple organ dysfunction syndrome (MODS)^6^. Thus, therapeutic restoration of intestinal barrier function, which requires the tight coordination of epithelial migration, proliferation and differentiation, is indispensable for intestinal I/R injury.

It has been reported that several nuclear receptors improved intestinal epithelial development and barrier function after injury^7–9^. Nuclear receptor-related protein 1 (nurr1), an orphan nuclear receptor, is a well-known transcription factor that participates in several cellular development processes, such as proliferation, differentiation and apoptosis^10,11^. We previously found that modulating nurr1 expression improves epithelial proliferation after intestinal I/R injury^12^. Increased epithelial cell proliferation contributes to enhance intestinal epithelial barrier function^13,14^. However, the effect of nurr1 on epithelial barrier function after intestinal I/R injury, as well as the mechanism by which nurr1 is modulated, require further investigation.

Recent research regarding nurr1 has demonstrated that its expression can be regulated by microRNAs (miRNAs) in some diseases^15–17^. miRNAs are a class of endogenous small non-coding RNAs of approximately 22 nucleotides in length that negatively modulate gene expression by promoting mRNA degradation or inhibiting transcript translation^18,19^. A growing number of studies have shown that miRNA modulation contributes to organ repair following a variety of events, including myocardial or cerebral ischemic injury^20,21^. However, miRNA-mediated gut epithelial restoration after intestinal I/R injury remains poorly understood. Based on the above findings regarding the role of nurr1, we hypothesized that miRNA modulation may improve intestinal epithelial proliferation and barrier function by targeting nurr1, thereby reducing intestinal I/R injury.

In this study, we investigated the regulatory role of nurr1 in epithelial barrier function after intestinal I/R injury. We performed microarray chip to identify the miRNAs that are differentially expressed between I/R-injured intestinal tissues and normal tissues and further screened miR-381-3p that targets nurr1. We speculate that miR-381-3p and nurr1 constitute an axis that regulates epithelial proliferation and barrier function after intestinal I/R injury. This study aimed to provide information regarding a potential strategy for the treatment of intestinal I/R injury.

## Results

### Nurr1 enhances intestinal epithelial regeneration and barrier function after intestinal I/R injury

**Fig. 1:**
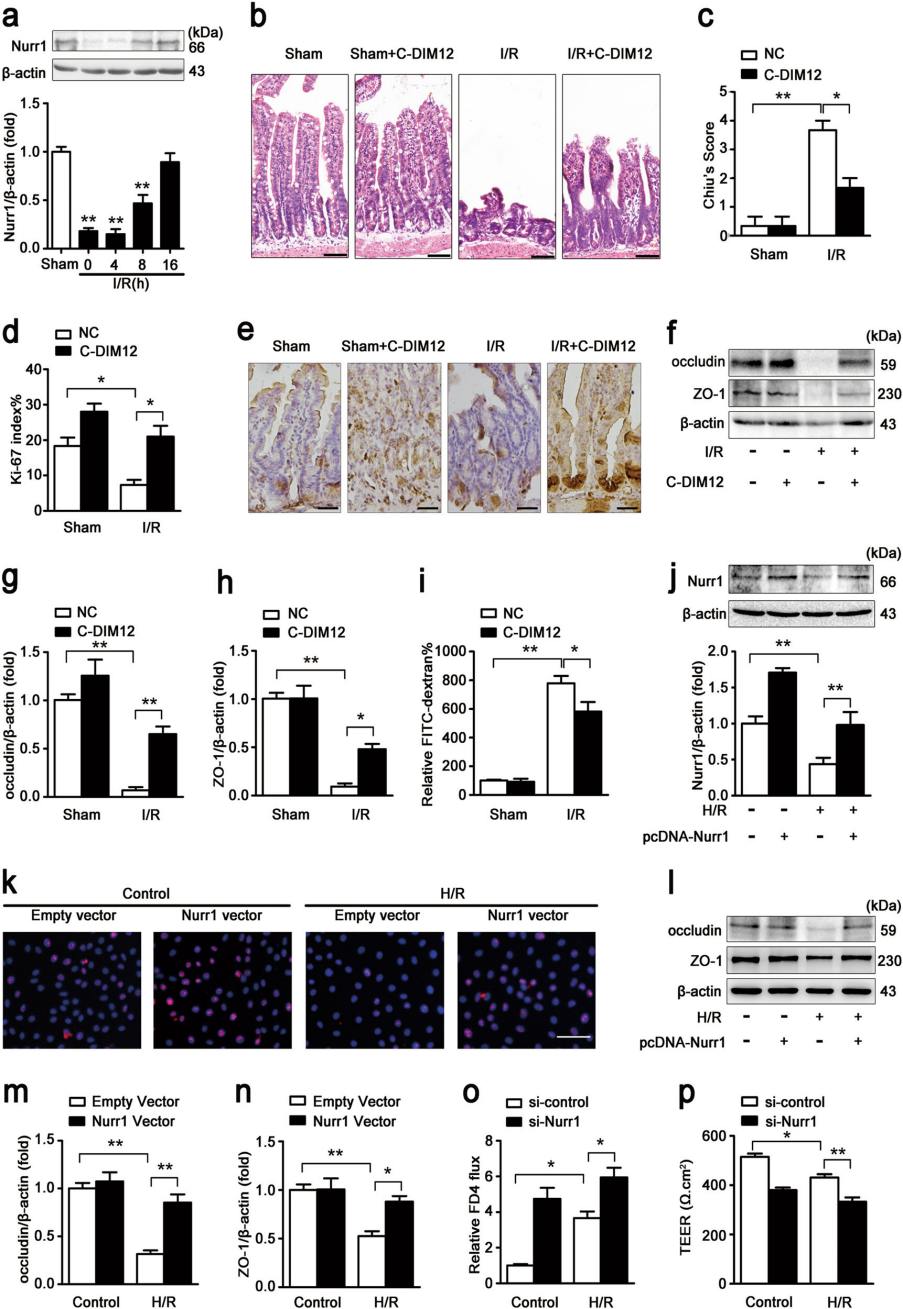
Nurr1 regulates intestinal restoration after I/R injury. **a** Mice were subjected to 45 min of intestinal ischemia followed by 0–16 h of reperfusion or sham surgery. I, ischemia; R, reperfusion; representative western blot showing nurr1 protein expression in the intestinal tissue lysates (*n* = 3 per group, **P < 0.01 versus sham group). **b-i** The mice were divided into the following four groups: a sham group, a sham + C-DIM12 group, an I/R group, and an I/R + C-DIM12 group (*n* = 8 per group). C-DIM12 (50 mg/kg) was orally gavaged at 4 h before surgery. The I/R times were 45/240 min, respectively. **b** and **c** Representative images of H&E-stained intestinal sections of mice from the above four groups. Intestinal injury was scored histopathologically (Chiu’s score) according to a scoring system. Scale bar = 100 μm. **d** and **e** Immunohistochemical staining for the Ki-67 antibody in intestinal tissues for proliferation analysis. Scale bar = 50 μm. **f-h** Representative western blot showing occludin and ZO-1 protein expression in the intestinal tissue lysates, *n* = 3 per group. **i** FITC-dextran intestinal epithelial paracellular permeability. **j-n** IEC-6 cells were transfected with plasmids encoding Nurr1 for 36 h and then incubated in hypoxic conditions for 6 h before being incubated in normoxic conditions for 6 h. **j** Representative western blot showing nurr1 protein expression, *n* = 3. **k** Immunofluorescence staining for the Ki-67 antibody in IEC-6 cells for proliferation analysis. Scale bar = 100 μm, *n* = 6. **l-n** Representative western blot showing occludin and ZO-1 protein expression, *n* = 3. **o** and **p** Caco-2 cells were infected with si-Nurr1 or its negative control (si-control) for 36 h and then exposed to hypoxic conditions for 12 h before being exposed to normoxic conditions 6 h. **o** Si-nurr1-induced changes in intestinal epithelial permeability, as measured by FITC-dextran permeability, *n* = 3. **p** The effect of si-Nurr1 on intestinal epithelial integrity was evaluated by TEER, *n* = 3. Data are representative of three independent experiments. *P < 0.05, **P < 0.01. The error bars describe the standard deviation

Western blotting was used to assess nurr1 expression at different reperfusion time points after intestinal ischemia. Intestinal nurr1 levels decreased significantly during the first 4 h after the onset of reperfusion and then progressively increased from 4–16 h after the onset of reperfusion, suggesting that nurr1 plays a critical role in regulating intestinal I/R injury (Fig. 1a). To validate the effect of nurr1 in vivo, we administered the nurr1 activator C-DIM12 to C57BL/6 mice. Compared with I/R group, intestinal histological injury was dramatically attenuated in the I/R + C-DIM12 group (Fig. 1b and c). Consistent with these findings, regeneration levels, as reflected by the numbers of Ki-67-positive cells, were higher in the I/R + C-DIM12 group than in the I/R group (Fig. 1d and e). Intestinal barrier functional restoration, a change reflected by increases in occludin and ZO-1 protein expression (Fig. 1f–h) and decreases in the FITC-dextran paracellular permeability, occurred in conjunction with the enhancement of epithelial regeneration (Fig. 1i).

Furthermore, in vitro pcDNA-Nurr1 transfection (Fig. 1j) increased Ki-67-labeled IEC-6 cell proliferation in hypoxia/reoxygenation (H/R; Fig. 1k) and occludin and ZO-1 protein expression (Fig. 1l–n). Conversely, RNAi-mediated Nurr1 knockdown decreased intestinal epithelial barrier function, a change reflected by increases in FITC-dextran paracellular permeability (Fig. 1o) and reductions in transepithelial electrical resistance (TEER; Fig. 1p) in Caco-2 cells. Consistent with the results of our previous study^12^, these results indicate that nurr1 improves intestinal epithelial proliferation and barrier function in I/R injury.

### miR-381-3p participates in regulating nurr1 expression

To explore the molecular mechanism underlying the effects of nurr1 down-regulation on H/R and I/R injury, we tested whether nurr1 is regulated by miRNA. Intestinal samples derived from mice subjected to intestinal I/R (*n* = 3) or sham surgery (*n* = 3) were collected and the miRNA expression profiles were determined by a miRNA microarray (GEO accession number: GSE83701). We found that 57 miRNAs were up-regulated, and 74 miRNAs were down-regulated (fold change ≥2, *P* < 0.05) in the I/R group compared with the sham group (Supplementary Table 1). It is well known that miRNAs negatively regulate the expression of specific genes by targeting their 3′-UTRs. Therefore, we used the miRNA prediction programs TargetScan^22^ (http://www.targetscan.org/) and miRWalk^23^ (http://zmf.umm.uni-heidelberg.de/apps/zmf/mirwalk) to determine that miR-381-3p, one of the 57 up-regulated miRNAs, targets nurr1.

**Fig. 2:**
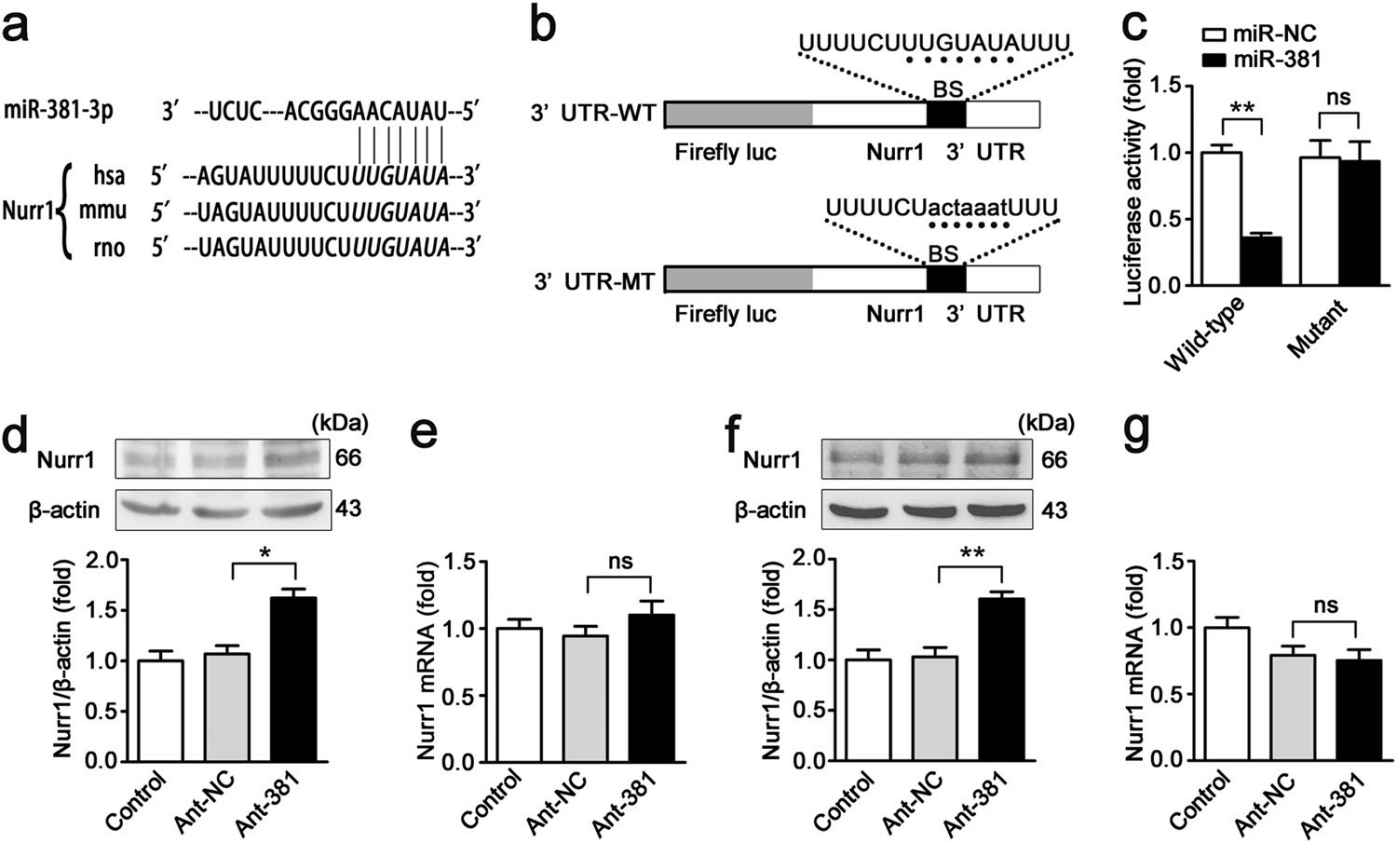
miR-381-3p negatively regulates nurr1 expression. **a** The miR-381-3p target sequence in the nurr1 3ʹ-UTR is conserved across various species. **b** The WT nurr1 3ʹ-UTR and the MT nurr1 3ʹ-UTR in the luciferase constructs. BS, binding site. **c** Caco-2 cells were infected with miR-381 or miR-NC and WT nurr1 3ʹ-UTR or MT nurr1 3ʹ-UTR, *n* = 3. **d-g** Caco-2 or IEC-6 cells were transfected with the ant-381 or the ant-NC. **d** Representative western blot showing nurr1 protein expression in Caco-2 cells, *n* = 3. **e** qRT-PCR showing nurr1 mRNA expression in Caco-2 cells, *n* = 6. **f** Representative western blot showing nurr1 protein expression in IEC-6 cells, *n* = 3. **g** qRT-PCR showing nurr1 mRNA expression in IEC-6 cells, *n* = 6. *P < 0.05, **P < 0.01. The error bars describe the standard deviation

According to the bioinformatics analysis, the miR-381-3p binding sequences in the 3′-UTR of nurr1 are highly conserved across several species, including humans, mice and rats (Fig. 2a, Supplementary Figure 1). We used a dual-luciferase assay system to further validate that miR-381-3p targets the 3′-UTR of nurr1. As demonstrated in Fig. 2b, the WT nurr1 3′-UTR exhibited low luciferase activity after miR-381-3p agomir (miR-381) transfection. However, the mutated nurr1 3′-UTR abolished the inhibitory effect of miR-381 (Fig. 2c). We then explored whether endogenous nurr1 expression is regulated by miR-381-3p modulation (Supplementary Figures 2a–c). miR-381-3p silencing up-regulated nurr1 protein levels but had no effect on mRNA expression in Caco-2 cells (Fig. 2d, e). These data showed that miR-381-3p represses nurr1 translation. Similar results were observed in the experiments involving IEC-6 cells (Fig. 2f, g). Taken together, these results indicate that miR-381-3p directly regulates nurr1 expression in the intestine.

### miR-381-3p inhibition increases intestinal epithelial proliferation and barrier function after H/R injury

**Fig. 3:**
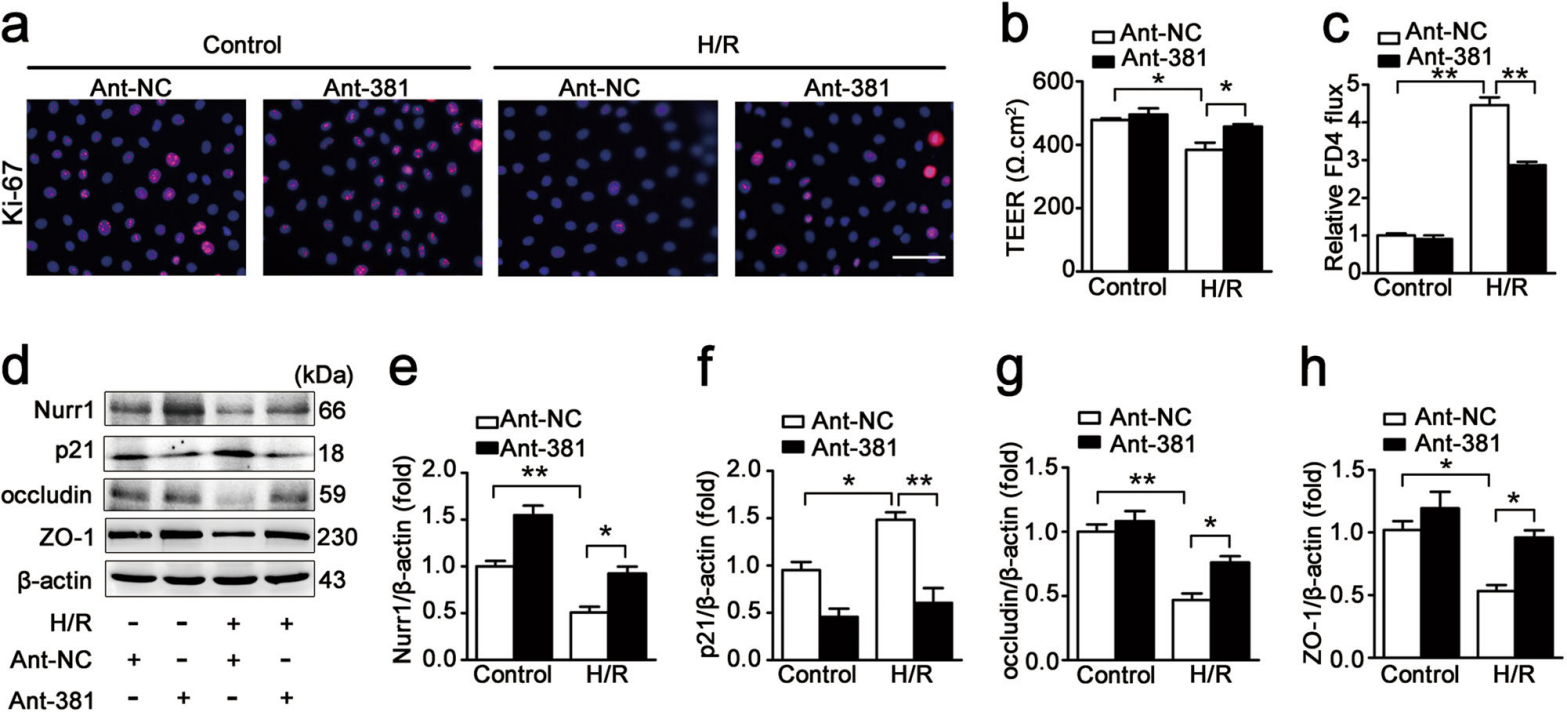
miR-381-3p inhibition promotes intestinal epithelial restoration after H/R injury. **a** Immunofluorescence staining for the Ki-67 antibody in IEC-6 cells for proliferation analysis. IEC-6 cells were infected with the ant-381 or the ant-NC for 36 h and then incubated under H/R conditions for 6/6 h, respectively. Scale bar = 100 μm, *n* = 6. **b** and **c** Caco-2 cells were transfected with the ant-381 or the ant-NC for 36 h and then exposed to hypoxic conditions for 12 h before being exposed to normoxic conditions for 6 h. **b** Effect of the ant-381 on TEER, *n* = 3. **c** Changes in FITC-dextran intestinal epithelial permeability in response to the ant-381, *n* = 3. **d-h** IEC-6 cells were infected with the ant-381 or the ant-NC for 36 h and then incubated under H/R conditions for 6/6 h, respectively. Representative western blot showing nurr1, p21, occludin, ZO-1 protein expression, *n* = 3. *P < 0.05, **P < 0.01. The error bars describe the standard deviation

We investigated the effect of miR-381-3p on intestinal epithelial cells exposed to H/R injury and the involvement of nurr1 signaling in this effect. miR-381-3p inhibition elevated Ki-67-labeled cell proliferation under H/R conditions (Fig. 3a). Consistently, miR-381-3p inhibition also restored TEER value (Fig. 3b) and attenuated the increases in FITC-dextran paracellular permeability (Fig. 3c) induced by H/R. Furthermore, western blotting revealed that miR-381-3p inhibition ameliorated the nurr1 down-regulation and p21 up-regulation induced by H/R (Fig. 3d–f). Tight junction protein (occludin and ZO-1) expression was clearly enhanced in the miR-381-3p inhibition group compared with the negative control group under H/R conditions (Fig. 3g, h). These data indicate that miR-381-3p inhibition increases intestinal epithelial proliferation and barrier function, and that the effect may be mediated by nurr1 signaling.

### miR-381-3p knockdown improves intestinal epithelial restoration and survival after intestinal I/R injury in mice

**Fig. 4:**
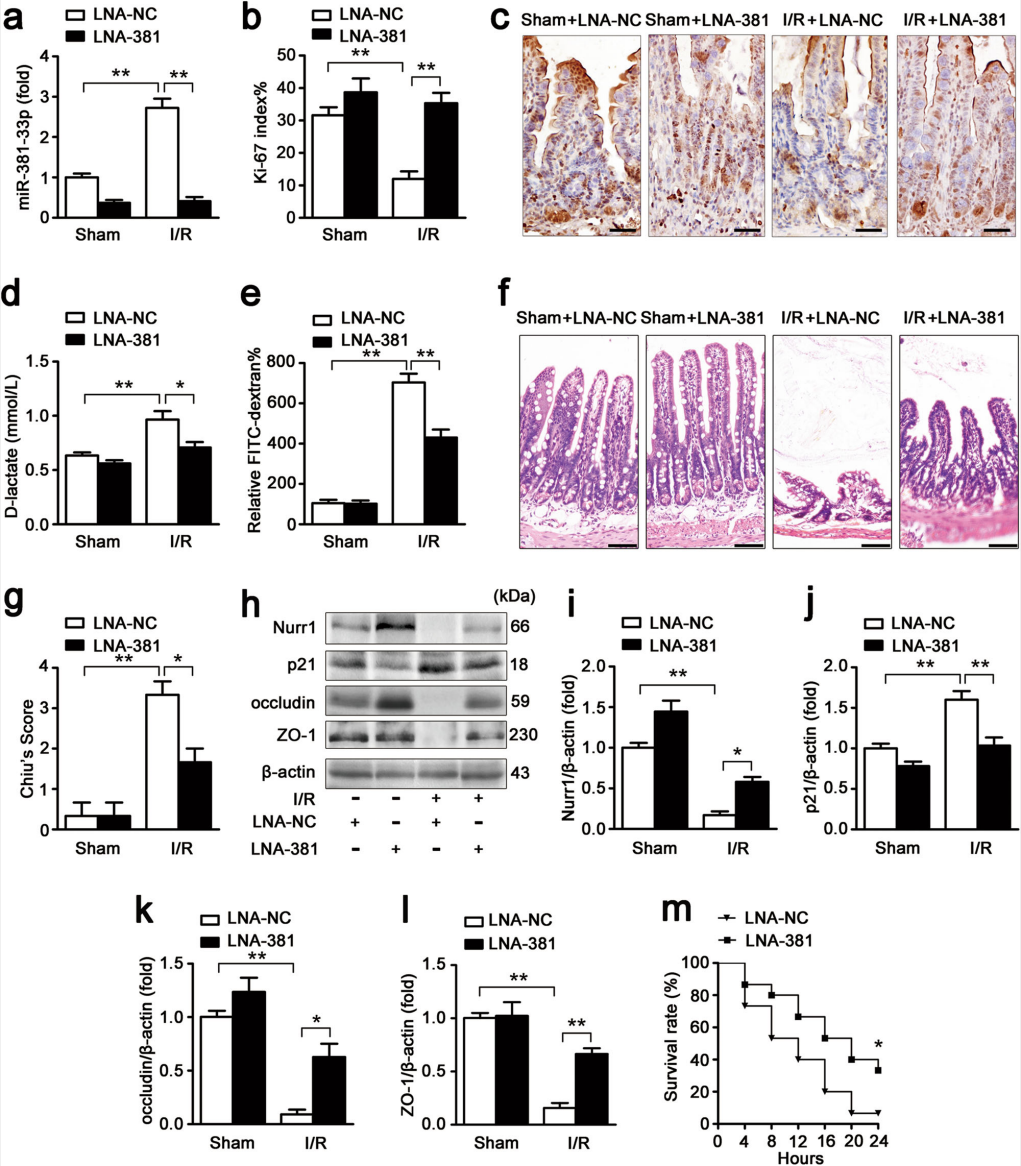
miR-381-3p inhibition improves intestinal barrier restoration after I/R injury in mice. The mice were divided into the following four groups: a sham + LNA-NC group, a sham + LNA-381 group, an I/R + LNA-NC group, an I/R + LNA-381 group (*n* = 8 per group). LNA-381 or LNA-NC (2 mg/kg) was administered by caudal vein injection at 12 h before surgery. The I/R times were 45/240 min, respectively. **a** qRT-PCR showing miR-381-3p expression, *n* = 8. **b** and **c** Immunohistochemical staining for the Ki-67 antibody in intestinal tissues for proliferation analysis. Scale bar = 50 μm, *n* = 6. **d** Serum d-lactate levels, *n* = 8. **e** FITC-dextran intestinal epithelial paracellular permeability, *n* = 8. **f** and **g** Representative images of H&E-stained intestinal sections of mice from the above four groups. Intestinal injury was scored histopathologically (Chiu’s score) according to a scoring system. Scale bar = 100 μm, *n* = 8. **h-l** Representative western blot showing nurr1, p21, occludin or ZO-1 protein expression in intestinal tissue, *n* = 3. **m** The survival rate of the mice, *n* = 15. *P < 0.05, **P < 0.01. The error bars describe the standard deviation

To determine the role of miR-381-3p in the regulation of the pathogenesis of intestinal I/R injury in vivo, we knocked down miR-381-3p expression in mice using LNA-381. As shown in Fig. 4a, LNA-381 dramatically attenuated the miR-381-3p up-regulation induced by intestinal I/R injury. Ki-67-labeled intestinal cell proliferation was enforced in the presence of LNA-381 (Fig. 4b, c). miR-381-3p knockdown reduced d-lactate levels (Fig. 4d) and FITC-dextran epithelial permeability (Fig. 4e) to attenuate the effect of I/R on intestinal barrier function. Intestinal histological injury was significantly mitigated after miR-381-3p silencing (Fig. 4f, g). Furthermore, LNA-381 preserved nurr1 expression and down-regulated p21 expression in mice subjected to intestinal I/R injury (Fig. 4h–j). Consequently, the expression of the tight junction proteins occludin and ZO-1 was enhanced after treatment with LNA-381 (Fig. 4k, l). In addition, we measured 24-h survival rates to test the long-term protective effects of LNA-381 under I/R injury. As shown in Fig. 4m, the overall survival rate was higher in the LNA-381 group than the LNA-NC group. Collectively, these findings support the notion that the knockdown of endogenous miR-381-3p restores epithelial barrier function and improves survival rates in intestinal I/R injury.

### miR-381-3p inhibition improves epithelial proliferation and barrier function after intestinal I/R injury by targeting nurr1

**Fig. 5:**
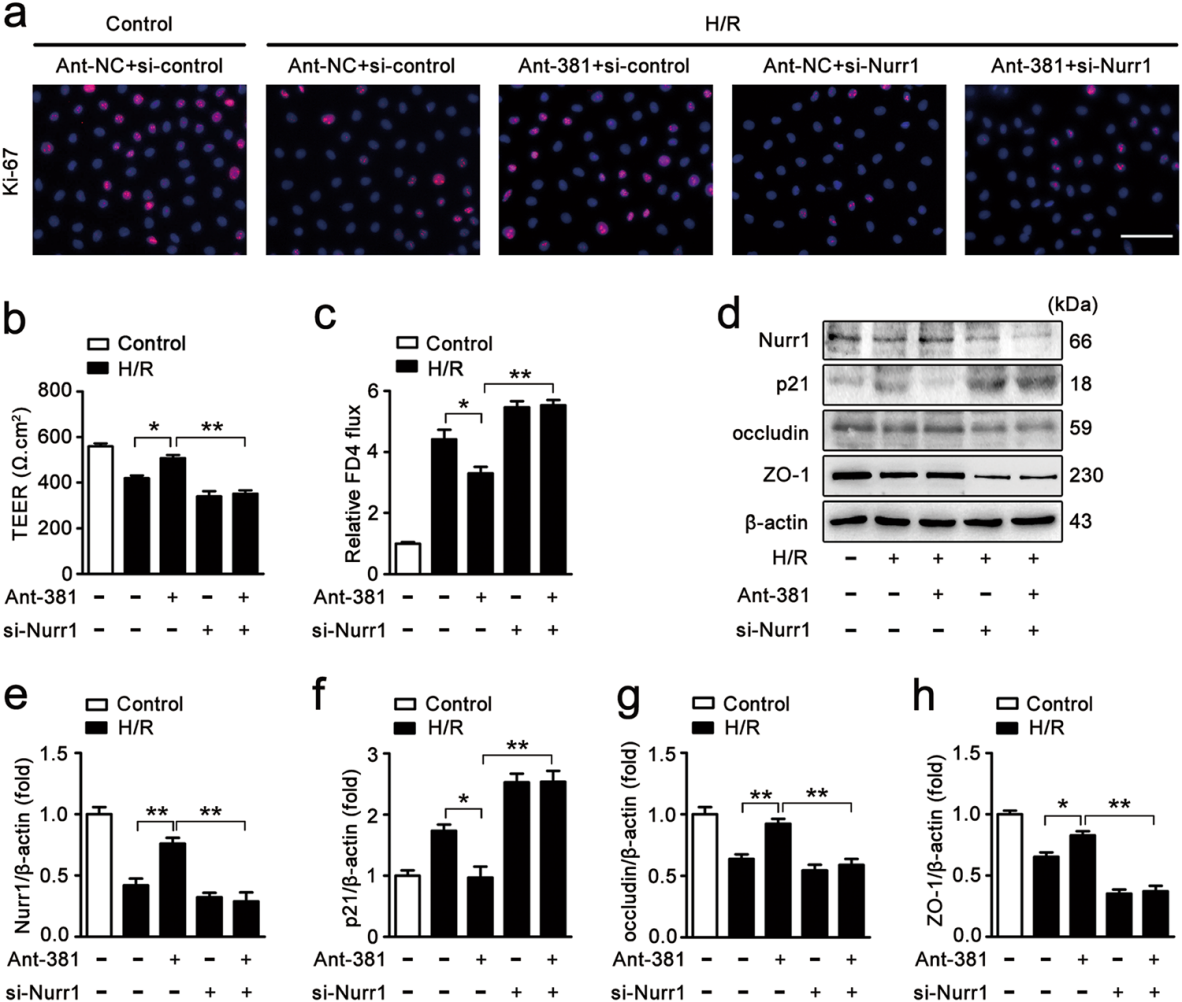
miR-381-3p inhibition provides protective effects by targeting nurr1. **a** Immunofluorescence staining for the Ki-67 antibody in IEC-6 cells for proliferation analysis. IEC-6 cells were infected with si-Nurr1 or si-control, transfected with the ant-381 or the ant-NC, and then incubated in H/R conditions for 6/6 h, respectively. Scale bar = 100 μm, *n* = 6. **b** and **c** Caco-2 cells were co-transfected with ant-381, si-Nurr1 or the corresponding negative control, and then exposed to H/R conditions for 12/6 h, respectively. **b** Effect of the ant-381 on TEER, *n* = 3. **c** Effect of the ant-381 on FITC-dextran intestinal epithelial permeability, *n* = 3. **d-h** IEC-6 cells were co-transfected with ant-381, si-Nurr1 or the corresponding negative control, and then incubated in H/R for 6/6 h, respectively. Representative western blot showing nurr1, p21, occludin, ZO-1 protein expression, *n* = 3. *P < 0.05, **P < 0.01. The error bars describe the standard deviation

The above experiments showed that miR-381-3p inhibition improved intestinal epithelial restoration and up-regulated nurr1 expression. Thus, we explored whether nurr1 plays an important role in the protective effects of miR-381-3p inhibition. The ant-381 elevated Ki-67-labeled cell proliferation and TEER and inhibited FITC-dextran paracellular permeability after H/R (Fig. 5a–c, groups 2 and 3). However, the protective effect of the ant-381 was abolished by the knockdown of nurr1 (Fig. 5a–c, groups 3 and 5). In addition, we also measured the expression of p21, a downstream target of nurr1^12^, to explore the potential mechanisms underlying the effects of miR-318-3p inhibition. As anticipated, p21 levels were markedly enhanced after H/R injury (Fig. 5f, groups 1 and 2). The ant-381 up-regulated nurr1 expression and inhibited p21 expression (Fig. 5d–f, groups 2 and 3), whereas nurr1 silencing abolished the effect of the ant-381 on p21 expression under H/R conditions (Fig. 5f, groups 3 and 5). Additionally, the ant-381 preserved occludin and ZO-1 expression after H/R; however, these effects were not observed in the nurr1-knockdown group (Fig. 5g, h). These findings suggest that miR-381-3p inhibition exerts its protective effects on intestinal I/R injury by modulating nurr1 expression.

### miR-381-3p knockdown attenuates mesenteric I/R-induced remote organ injury

**Fig. 6:**
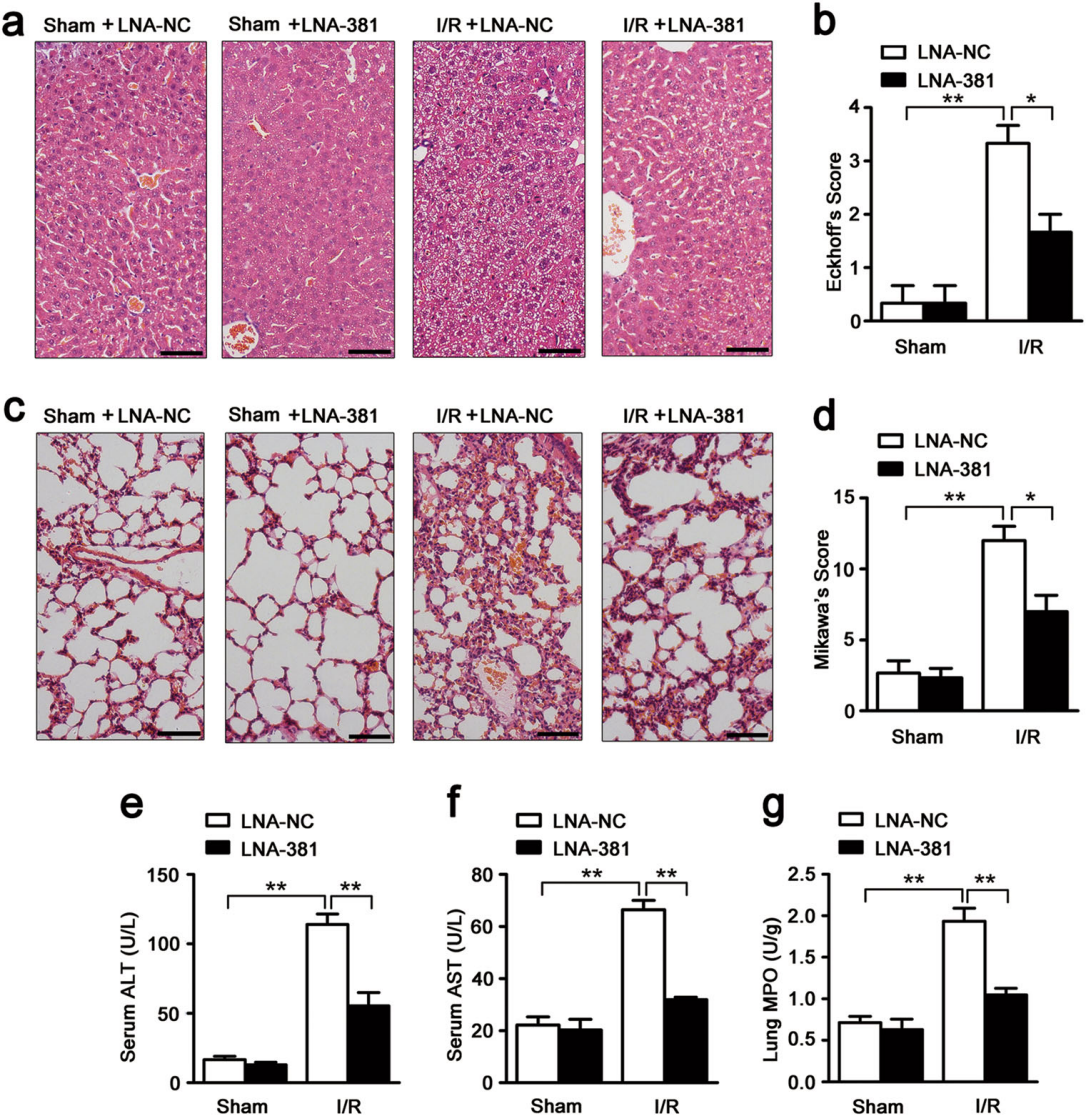
miR-381-3p inhibition attenuates intestinal I/R induced liver and lung injury. The mice were divided into the following four groups: a sham + LNA-NC group, a sham + LNA-381 group, an I/R + LNA-NC group, an I/R + LNA-381 group (*n* = 8). LNA-381 or LNA-NC (2 mg/kg) was injected via the caudal vein at 12 h before surgery. The I/R times were 45/240 min, respectively. **a** and **b** Representative images of H&E-stained hepatic sections from mice. Hepatic injury was scored histopathologically (Eckhoff’s score) according to a scoring system. Scale bar = 100 μm. **c** and **d** Representative images of H&E-stained lung sections from mice. Lung injury was scored histopathologically (Mikawa’s score) according to a scoring system. Scale bar = 100 μm. **e** Serum ALT levels. **f** Serum AST levels. **g** MPO activity in the lung. *n* = 8. *P < 0.05, **P < 0.01. The error bars describe the standard deviation

Intestinal I/R is invariably followed by remote organ injury, which plays a critical role in prognosis^24^. Therefore, liver and lung tissue injury was examined to evaluate the protective effect of miR-381-3p knockdown on remote organ injury. As illustrated in Fig. 6a–d, miR-381-3p silencing clearly mitigated intestinal I/R-induced liver and lung histological injury. Intestinal I/R-induced augmentations of ALT and AST levels were significantly reduced in LNA-381-pretreated mice compared with LNA-NC-pretreated mice (Fig. 6e, f). Similarly, intestinal I/R-induced lung neutrophilic infiltration (as demonstrated by MPO activity) was markedly attenuated after LNA-381 administration (Fig. 6g). These results indicate that miR-381-3p knockdown ameliorates the remote hepatic and pulmonary damage induced by intestinal I/R.

### miR-381-3p, nurr1 and tight junction protein expression in the ischemic intestine of clinical patients

**Fig. 7:**
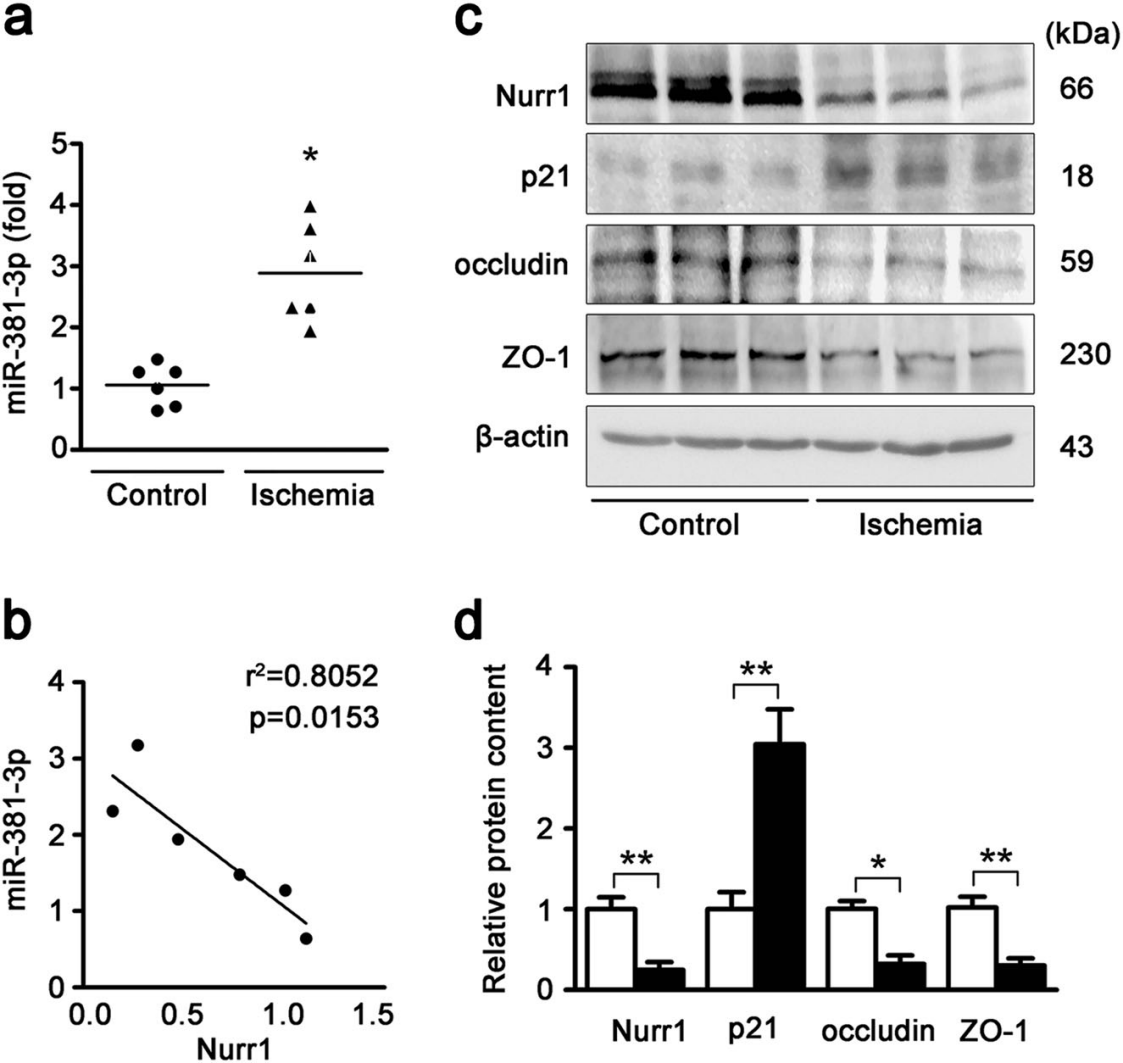
miR-381-3p, nurr1 and tight junction protein expression levels in the ischemic intestine of clinical patients. **a** qRT-PCR showing miR-381-3p expression in normal and ischemic human intestines, *n* = 6. **b** The association between miR-381-3p expression and Nurr1 protein expression (*r*^2^ = 0.8052, *p* = 0.0153). **c** and **d** Representative western blot showing nurr1, p21, occludin or ZO-1 protein expression in the intestine, *n* = 3. *P < 0.05, **P < 0.01. The error bars describe the standard deviation

To investigate the clinical relevance of miR-381-3p in intestinal I/R injury, we measured the expression of miR-381-3p and associated genes in ischemic intestinal tissues and normal tissues from clinical patients. Intestinal ischemia contributed to the elevation of miR-381-3p expression (Fig. 7a) and the reduction of nurr1 expression (Fig. 7c, d). Thus, miR-381-3p expression was inversely correlated with nurr1 expression in ischemic gut tissue (Fig. 7b). Furthermore, occludin and ZO-1 down-regulation under ischemia suggested that miR-381-3p plays a role in regulating epithelial barrier function (Fig. 7c, d). Collectively, these findings support the idea that miR-381-3p modulates nurr1 signaling, thereby affecting intestinal ischemia injury in clinical patients.

## Discussion

In this study, we identified a miRNA-regulated signaling pathway that alleviates intestinal I/R injury. We made the following observations: (1) nurr1 regulated intestinal epithelial barrier function after intestinal I/R injury; (2) among the miRNAs differentially expressed between ischemic and normal tissues, miR-381-3p directly suppressed nurr1 translation; (3) miR-381-3p inhibition ameliorated intestinal I/R injury in vitro and in vivo, attenuated remote organ injury and improved survival; (4) miR-381-3p inhibition exerted protective effects on intestinal I/R injury by targeting nurr1. To the best of our knowledge, this study showed for the first time that miRNA-mediated restoration of intestinal epithelial barrier function attenuates intestinal I/R injury.

Loss of intestinal barrier function caused by enterocyte disruption or cell death is a particularly dangerous complication of intestinal I/R injury, as the complication may lead to life-threatening bacterial translocation from the gut, numerous secondary organ injuries, multi-system organ failure and death^25^. Intestinal barrier repair is based on the tight coordination of cell migration, proliferation and differentiation^26^. Enterocytes migrate from the crypt base to villi while differentiating into enterocytes, goblet cells and enteroendocrine cells during intestinal epithelial development^27,28^. Cell proliferation within the crypt is the primary force driving cell migration along the villus^29^. Therefore, increased epithelial cell proliferation contributes to intestinal barrier restoration^13,14^. We recently found that nurr1 promotes epithelial proliferation after intestinal I/R injury^12^. In the current study, we found that nurr1 protein expression reached the valley value at 4 h after the onset of reperfusion. Concomitant decreases in gut epithelial proliferation and barrier function were observed. Gain- and loss-of-function studies revealed that nurr1 overexpression enhanced epithelial proliferation and barrier function after I/R injury. However, nurr1 silencing had the opposite effect. Thus, these data demonstrate that nurr1 improves epithelial barrier function after intestinal I/R injury.

An increasing number of studies have shown that nurr1 is involved in ischemia/reperfusion injury. Nurr1 mRNA is reportedly up-regulated during liver or kidney I/R injury, indicating that nurr1 may be associated with the aggravation of I/R injury^30,31^. However, Hossmann et al. and our team suggested that nurr1 protein expression is down-regulated during stroke or intestinal I/R injury^12,32^. In addition, we found that nurr1 mediated epithelial proliferation in intestinal I/R injury^12^. Here, the nurr1 activator C-DIM12 significantly attenuated intestinal I/R injury in mice, a change reflected by improvements in intestinal histological injury, increases in tight junction protein expression and decreases in intestinal permeability. These data indicate that nurr1 mitigates intestinal I/R injury. We speculate that the different roles of nurr1 in I/R injury may be tissue type dependent.

Recent reports have shown that miRNAs regulate key pathogenic events and affect intestinal I/R injury^33,34^. However, few reports have examined whether miRNAs mitigate intestinal I/R injury by restoring epithelial barrier function. To thoroughly clarify the effects of miRNA on epithelial barrier repair after intestinal I/R injury, we utilized a miRNA microarray platform to identify 131 miRNAs that are differentially expressed between the I/R group and the sham group. Based on our findings regarding the effects of nurr1 on intestinal barrier function, we used bioinformatics analysis to determine miR-381-3p that can target nurr1. The miR-381-3p binding sequences in the nurr1 3′-UTR are highly conserved across various species. Gain-of-function and loss-of-function studies demonstrated that miR-381-3p directly repressed nurr1 translation. We also showed that miR-381-3p was involved in regulating gut barrier function after I/R. In vitro, miR-381-p inhibition enhanced intestinal epithelial proliferation and barrier function by up-regulating nurr1 under H/R conditions. In vivo, LNA-mediated miR-381-p knockdown prevented intestinal I/R injury by improving epithelial proliferation and tightening the intestinal epithelium. In addition, our study demonstrated that nurr1-mediated p21 repression stimulates intestinal epithelial cell proliferation^12^. p21 suppression restores the barrier function of skin suffering from the loss of Dicer function by regulating cellular proliferation and differentiation^35^. In this study, p21 was significantly down-regulated when miR-381-3p was inhibited to promote nurr1-mediated epithelial barrier function after intestinal I/R injury. These data suggest that miR-381-3p inhibition improves epithelial proliferation and barrier function by modulating the nurr1/p21 pathway, and then attenuates intestinal I/R injury.

Intestinal I/R injury-induced MODS is a leading cause of death in critically ill ICU patients^36,37^. As demonstrated in this study, mesenteric I/R injury aggravates lung and liver histopathological damage, as well as pulmonary neutrophil infiltration and liver dysfunction. Interesting, we found that miR-381-3p knockdown contributes to the attenuation of lung and liver injury and improvements in survival after mesenteric I/R. These results suggest that miR-381-3p is an important target for the treatment of intestinal I/R-induced MODS.

LNA oligonucleotides are conformational RNA analogs that have an unprecedented affinity for and bind specifically to complementary RNA, leading to selective miRNA silencing^38,39^. A LNA-modified inhibitor of miR-122 (Miravirsen) was used in phase 2 clinical trials of treatments for HCV infection and induced dose-dependent reductions in HCV RNA levels that endured beyond the end of active therapy^40^. In this study, LNA-381 was injected into C57 mice to specifically reduce miR-381-3p expression in vivo without inducing hepatotoxicity. We observed that LNA-381 attenuated local intestinal damage and remote organ injury and improved survival in mice subjected to mesenteric I/R. These findings support that LNA-381 may be a therapeutic intervention for clinical patients with intestinal I/R.

Previous studies have shown that miR-381-3p may be a target for the treatment of patients with TB or HIV-associated neurocognitive disorders^41,42^. To determine the clinical significance of miR-381-3p in patients with intestinal I/R injury, we measured the expression of miR-381-3p and associated proteins in ischemic intestinal tissues and normal tissues from clinical patients. The inverse relationship between miR-381-3p and nurr1 expression was confirmed through analysis of the intestinal tissue samples. In addition, the finding that intestinal ischemia down-regulated the expression of tight junction proteins suggested that miR-381-3p plays a crucial role in intestinal barrier function. To validate the regulatory role of miR-381-3p in clinical patients with intestinal ischemia, we need to collect enough samples to analyze its function in such patients.

In summary, we found that nurr1 enhanced intestinal barrier function after intestinal I/R injury. Using microarray and bioinformatics analysis, we found that miR-381-3p targets nurr1. Furthermore, our study demonstrated that miR-381-3p inhibition improves epithelial proliferation and barrier function by targeting nurr1 signaling, thereby reducing Intestinal I/R injury. This may provide a new miRNA-based strategy for the treatment of intestinal I/R-related clinical conditions.

## Materials and methods

### Murine model of intestinal I/R

Young male C57BL/6 mice (aged 8 weeks) were purchased from the Animal Center of Dalian Medical University (Dalian, China). The mice were housed in specific pathogen-free conditions at a constant temperature (22 ± 2 °C) and under a 12-h light/dark cycle. The mice were fed standard food and water and were given 1 week to acclimate to their environment before being used in the study. The mice were randomly divided into the following four groups: a sham group, a sham + 1,1-bis(39-indolyl)-1-(p-chlorophenyl) methane (C-DIM12) group, an I/R group, and an I/R + C-DIM12 group. All mice were anesthetized via an intraperitoneal injection of pentobarbital (50 mg/kg body weight). Midline laparotomy was performed, and the superior mesenteric artery was identified, isolated and clamped. After 45 min of ischemia, the vascular clamp was removed from the artery to allow reperfusion. The mice used for the survival study were anesthetized with buprenorphine (0.1 mg/kg, IP, every 12 h)^43^. Corn oil was used to dissolve C-DIM12 (Selleck, Houston, USA). C-DIM12 (50 mg/kg body weight) was administered by gavage at 4 h prior to surgery, in accordance with existing pharmacokinetic data^44^. After 4 h of reperfusion, intestinal tissue samples and blood samples were harvested for various experimental evaluations required for this study. All procedures were performed in accordance with the guidelines for the care and use of medical laboratory animals. This study was approved by the Institutional Ethics Committee of Dalian Medical University (Dalian, China).

### Cell culture and H/R

Caco2 cells and IEC-6 cells were obtained from American Type Culture Collection (Manassas, VA, USA) and grown in Dulbecco's modified eagle medium supplemented with 10% fetal bovine serum, 1% non-essential amino acids and 1% glutamine. IEC-6 cells were also treated with 0.1 unit/ml bovine insulin. The cultures were maintained at 37 °C in a humidified atmosphere of 5% CO_2_. To induce hypoxia, we incubated the Caco2 and IEC-6 cells in a microaerophilic system (Thermo, Waltham, MA) containing 1% O_2_ and 5% CO_2_ balanced with 94% N_2_ gas for 12 h and 6 h, respectively. The cells were then reoxygenated under normoxic conditions for 6 h.

### Transfection of pcDNA-Nurr1, small interfering RNA (siRNA), agomirs and antagomirs

Plasmids encoding nurr1 were used to over-express the protein. We previously described a siRNA that targets nurr1. A nonspecific scrambled siRNA duplex was used as a control. A chemically modified antagomir complementary to miR-381-3p was used to inhibit miR-381-3p expression, while an agomir was used to increase miR-381-3p expression. The plasmids, siRNAs, agomirs, antagomirs and corresponding negative control oligonucleotides were purchased from GenePharma (Shanghai, China). The sequences of the oligonucleotides are shown in Table 1. The cells were transfected with the oligonucleotides using Lipofectamine 3000, according to the manufacturer’s protocols.

**Table 1:** Sequences of antagomirs, agomirs, LNAs, siRNAs and primers

Name	Sequences (5′–3′)
miR-381-3p antagomir (human)	ACAGAGAGCUUGCCCUUGUAUA
miR-381-3p antagomir (rat)	AGAGCUUGCCCUUGUAUA
antagomir negative control	CAGUACUUUUGUGUAGUACAA
miR-381-3p agomir (human)	UAUACAAGGGCAAGCUCUCUGU AGAGAGCUUGCCCUUGUAUAUU
agomir negative control	UUCUCCGAACGUGUCACGUTT ACGUGACACGUUCGGAGAATT
LNA-381 (mouse)	GAGCUUGCCCUUGUAU
LNA-NC (mouse)	TACGTCTATACGCCCA
nurr1 siRNA (human)	GGCUUGUAAAUUUACCCAATT UUGGGUAAAUUUACAAGCCTT
nurr1 siRNA (rat)	CCUCACCAACACUGAAAUUtt AAUUUCAGUGUUGGUGAGGtc
siRNA negative control	UUCUCCGAACGUGUCACGUTT ACGUGACACGUUCGGAGAATT
nurr1 (human) Forward Reverse	GGCCGGAGAGGTCGTTTGCC AGGGTTCGCCTGGAACCTGGA
nurr1 (rat) Forward Reverse	GGGCTCAAGGAACCCAAGAG GCAAAGGGTGCGAAGTTCTG

### miRNAs microarray analysis

The microarray analysis for miRNA profiling was conducted by the miRCURY LNA Array system (Exiqon, Vedbaek, Denmark). The total RNA was extracted from intestinal samples of mice subjected to intestinal I/R or sham surgery using Trizol (Invitrogen) according to the instructions provided by the manufacturer. The quality and quantity of RNA samples were assessed by using nanodrop spectrophotometer (ND-1000, Nanodrop Technologies). RNA samples were labeled with an Exiqon miRCURY™ Hy3™/Hy5™ Power labeling kit and hybridized on a miRCURYTM LNA Array station. Scanning was performed with an Axon GenePix 4000B microarray scanner (Axon Instruments, Foster City, CA). Scanned images were then imported into GenePix Pro 6.0 software (Axon) for grid alignment and data extraction. Replicated miRNAs were averaged and miRNAs that intensities ≥30 in all samples were chosen for calculating normalization factor. Expressed data were normalized using the Median normalization. Discriminant miRNAs and differences between groups were analyzed using Bayes moderated *t*-test (limma) with Benjamini Hochberg false discovery rate at *P* < 0.05, unless otherwise specified. A two-fold cut-off was applied to select up-regulated and down-regulated miRNAs.

### Luciferase activity assay

Plasmids containing the wild-type miR-381-3p 3′-untranslated region (3′-UTR-WT) or the corresponding mutant sequence (3′-UTR-mut) were purchased from GenePharma. Plasmid DNA and ago-381 or ago-NC were co-transfected into cells seeded in 24-well plates using Lipofectamine 3000. The Caco-2 cells were evaluated with the Dual-Luciferase Reporter Assay Kit (TransGen, Beijing, China) after 36 h of transfection. Luciferase activity was measured using a Dual-Light Chemiluminescent Reporter Gene Assay System (Berthold, Germany) normalized to Renilla luciferase activity.

### Immunofluorescence

IEC-6 cells were seeded on chamber-slides, fixed in ice-cold 4% paraformaldehyde for 15 min, permeabilized using 0.1% Triton X-100 for 10 min and then blocked with 3% BSA in PBS for 1 h at 37 °C. The cells were subsequently incubated with Ki-67 (1:200 Abcam, UK) antibodies overnight at 4 °C, after which they were incubated with Alexa flour 594 secondary antibodies (Invitrogen Life Technologies, Carlsbad, CA, USA) for 1h at room temperature and treated with DAPI (1 μg/μl for 10 min) at room temperature to stain the nuclei. The resulting immunofluorescence was examined using an Olympus fluorescence microscope.

### Transepithelial electrical resistance (TEER) assay

To assess intestinal barrier function in vitro, we used 24-well transwell system plates (0.4-µm pore size, Costar Incorporated Corning, NY) to perform TEER assay, as described previously^45^. Because IEC-6 cells rarely form a tight monolayer, we used Caco-2 cells to study intestinal barrier function by TEER assay^45,46^. Briefly, 0.3 ml of Caco-2 cells were seeded in the upper chamber of the transwell system at a density of 1 × 10^5^ cells/ml. The apical one bathed in the basal chamber with 0.7 ml medium for 20 days. TEER was measured daily by an epithelial volt ohmmeter (WPI, Sarasota, FL, USA). The medium was refreshed every day. The Caco-2 cells were transfected with antagomirs or siRNA during a steady monolayer state. TEER was calculated by the following formula: TEER = (R1 − R0) × *A* Ω cm^2^, where R1 is the background resistance, R0 is the collagen layer and membrane insert resistance, and A is the insert membrane area. The mean TEER was calculated using data from three experiments.

### In vivo knockdown of miR-381-3p

To inhibit miR-381-3p expression in vivo, we injected mice with 2 mg/kg locked nucleic acid-modified antisense oligonucleotides targeting miR-381-3p (LNA-381) or LNA-NC oligonucleotides via the tail vein at 12 h before ischemia. The LNA oligonucleotides were obtained from Exiqon Vedbaek, Denmark. Their sequences are shown in Table 1.

### Histopathology and immunohistochemistry

To evaluate intestinal, hepatic and pulmonary injury after intestinal I/R, we divided paraffin-embedded tissue samples into 4-μm sections, which we stained with hematoxylin and eosin (H&E). The intestinal, hepatic and pulmonary pathological scores were graded based on Chiu,^47^ Eckhoff^48^ and Mikawa,^49^ respectively. Mouse small intestinal tissue sections were blocked with 5% goat serum, after which they were incubated with anti-Ki-67 antibodies overnight at 4 °C. An ABC kit and DAB substrates were used for Ki-67. The sections were then counterstained by hematoxylin. The IHC results were examined using a fluorescence microscope.

### Intestinal permeability assay

Intestinal barrier permeability was measured by 4.4-kDa fluorescein isothiocyanate FITC-dextran (FD4, Sigma-Aldrich, USA) fluorescence intensity. We used Caco-2 cells instead of IEC-6 cells because IEC-6 cells are unable to form a tight monolayer^46^. We added 1.0 mg/ml FD-4 to the apical side of each monolayer after H/R injury, after which a 100-μl sample was taken from the basolateral chamber and assayed for fluorescence using an Enspire2300 microplate reader (excitation, 480 nm; emission, 520 nm). Two-hundred microliters of PBS containing 25 mg/ml FD-4 was gavaged orally during ischemia, as described in a previous study. The 100-μl blood sample was collected after the reperfusion period^50^. The fluorescence intensity of each sample was measured with an Enspire2300 microplate reader (excitation, 480 nm; emission, 520 nm).

### Real-time PCR

miRNA was isolated from the cells with TRIzol (Takara, Dalian, China). We detected miR-381-3p with a Bulge-Loop miRNA qRT-PCR Primer Set (Ribobio Co.), according to the manufacturer’s instructions. The expression levels of the miRNA were normalized to those of U6 snRNAs, which served as an endogenous control. Total RNA was extracted by TRIzol reagent and reversed transcribed with a Transcript All-in-one SuperMix for qPCR Kit (TransGen, Beijing, China). Nurr1 mRNA expression was quantified using a SYBR Real-time PCR Kit (TransGen, Beijing, China). Specific primers were produced by Invitrogen (Ribobio Co.). The sequences of the primers are listed in Table 1. Nurr1 mRNA levels were normalized to β-actin mRNA levels.

### Western blotting

Intestinal tissue and cell protein were extracted according to the manufacturer’s instructions (KeyGEN Biotech, Nanjing, China). Western blotting was performed with primary antibodies against nurr1, p21 (Abcam, Cambridge, UK), ZO-1, occludin (Proteintech, Wuhan, China), and β-actin (ZSGB-BIO, Beijing, China) and horseradish peroxidase (HRP)-conjugated secondary antibodies (ZSGB-BIO, Beijing, China). Protein levels were analyzed using Gel-Pro Analyzer Version 4.0 (Media Cybernetics, MD, USA).

### d-lactate, ALT, AST and MPO activity assays

Serum d-lactate content was assayed using an enzyme-linked immunosorbent assay kit (J&L Biological, Shanghai, China), according to the manufacturer’s instructions. Pulmonary MPO activity levels and serum ALT and AST levels were measured using commercial assay kits (Nanjing Jiancheng Corp, Nanjing, China), according to the manufacturer’s protocols.

### Acquisition of clinical specimens

The collection and use of human intestinal samples were approved by the local ethics committee at Dalian Medical University. Patient intestinal samples were obtained after the patients provided informed written consent. Ischemic gut segments, which were assigned to the model group, and the margins of the resected gut segments, which were assigned to the control group, were obtained from patients who underwent surgery for acute superior mesenteric artery emboli or intestinal hernia strangulation in the Department of Acute Abdominal Surgery of the Second Affiliated Hospital of Dalian Medical University. The intestinal samples were immediately frozen in liquid nitrogen and stored at −80 °C until analysis.

### Statistical analysis

All values are expressed as the mean ± SD. Data with normal distributions were compared using one-way analysis of variance followed by the Student–Newman–Keuls test. The survival study results were analyzed using the Kaplan–Meier method. A two-tailed Student’s *t*-test was used to compare means between two groups. At least three independent experiments were performed to confirm the results. Statistical analysis was performed using GraphPad Prism 5.0 (GraphPad Prism Software, La Jolla, CA, USA). *P* < 0.05 was considered statistically significant.

## Electronic supplementary material

Supplementary Figure 1(DOC 83 kb)

Supplementary Figure 2(DOC 264 kb)

Supplementary Table 1(DOC 175 kb)

## References

[1] Kong SE, Blennerhassett LR, Heel KA, Mccauley RD, Hall JC (1998). Ischaemia‐reperfusion injury to the intestine. Aust. N.Z. J. Surg..

[2] Acosta S, Björck M (2014). Modern treatment of acute mesenteric ischaemia. Br. J. Surg..

[3] Stone JR, Wilkins LR (2015). Acute mesenteric ischemia. Tech. Vasc. Interv. Radiol..

[4] Gibot S (2008). Effects of the TREM-1 pathway modulation during mesenteric ischemia-reperfusion in rats. Crit. Care Med..

[5] Jin X, Zimmers TA, Zhang Z, Pierce RH, Koniaris LG (2010). Interleukin-6 is an important in vivo inhibitor of intestinal epithelial cell death in mice. Gut.

[6] Kannan L (2013). R-spondin3 prevents mesenteric ischemia/reperfusion-induced tissue damage by tightening endothelium and preventing vascular leakage. Proc. Natl. Acad. Sci. USA.

[7] Meckel K (2016). Serum 25-hydroxyvitamin D concentration is inversely associated with mucosal inflammation in patients with ulcerative colitis. Am. J. Clin. Nutr..

[8] Klepsch, V. et al. Nuclear orphan receptor NR2F6 as a safeguard against experimental murine colitis. *Gut* [Epub ahead of print] 10.1136/gutjnl-2017-314241 (2017). 10.1136/gutjnl-2016-313466 PMC6204953 28779026

[9] Gadaleta RM (2011). Farnesoid X receptor activation inhibits inflammation and preserves the intestinal barrier in inflammatory bowel disease. Gut.

[10] Chen P (2016). Adenovirus-mediated expression of orphan nuclear receptor NR4A2 targeting hepatic stellate cell attenuates liver fibrosis in rats. Sci. Rep..

[11] Llopis S (2013). Dichotomous roles for the orphan nuclear receptor NURR1 in breast cancer. BMC Cancer.

[12] Guo Z (2016). Nurr1 promotes intestinal regeneration after ischemia/reperfusion injury by inhibiting the expression ofp21 (Waf1/Cip1). J. Mol. Med..

[13] Shim S (2017). Rebamipide ameliorates radiation-induced intestinal injury in a mouse model. Toxicol. Appl. Pharmacol..

[14] Nan X (2016). Hsa-miRNA-31 regulates epithelial cell barrier function by inhibiting TNFSF15 expression. Cell Mol. Biol. (Noisy-Le.-Grand.).

[15] Yan W, Chen ZY, Chen JQ, Chen HM (2016). BMP2 promotes the differentiation of neural stem cells into dopaminergic neurons in vitro via miR-145-mediated upregulation of Nurr1 expression. Am. J. Transl. Res..

[16] Yang D (2012). miR-132 regulates the differentiation of dopamine neurons by directly targeting Nurr1 expression. J. Cell Sci..

[17] Wu S (2015). MicroRNA-132 promotes estradiol synthesis in ovarian granulosa cells via translational repression of Nurr1. Reprod. Biol. Endocrinol..

[18] Bartel DP (2009). MicroRNAs: target recognition and regulatory functions. Cell.

[19] Lee Y (2003). The nuclear RNase III Drosha initiates microRNA processing. Nature.

[20] Lesizza P (2017). Single-dose intracardiac Injection of pro-regenerative MicroRNAs improves cardiac function after myocardial infarction. Circ. Res..

[21] Yang J, Zhang X, Chen X, Lei W, Yang G (2017). Exosome mediated delivery of miR-124 promotes neurogenesis after ischemia. Mol. Ther. Nucleic Acids.

[22] Enright AJ (2003). MicroRNA targets in Drosophila. Genome. Biol..

[23] Dweep H, Sticht C, Pandey P, Gretz N (2011). miRWalk-Database: prediction of possible miRNA binding sites by “walking” the genes of three genomes. J. Biomed. Inform..

[24] Moraes LB (2008). Gut ischemia/reperfusion induced acute lung injury is an alveolar macrophage dependent event. J. Trauma.

[25] Kannan KB (2011). Hypoxia-inducible factor plays a gut-injurious role in intestinal ischemia reperfusion injury. Am. J. Physiol. Gastrointest. Liver Physiol..

[26] Iizuka M, Konno S (2011). Wound healing of intestinal epithelial cells. World J. Gastroenterol..

[27] Sun X, Yang Q, Rogers CJ, Du M, Zhu MJ (2017). AMPK improves gut epithelial differentiation and barrier function via regulating Cdx2 expression. Cell Death Differ..

[28] Clevers H, Batlle E (2013). SnapShot: the intestinal crypt. Cell.

[29] Parker A (2017). Cell proliferation within small intestinal crypts is the principal driving force for cell migration on villi. FASEB J..

[30] Ohkubo T (2002). Early induction of nerve growth factor-induced genes after liver resection–reperfusion injury. J. Hepatol..

[31] Balasubramanian S, Jansen M, Valerius MT, Humphreys BD, Strom TB (2012). Orphan nuclear receptor Nur77 promotes acute kidney injury and renal epithelial apoptosis. J. Am. Soc. Nephrol..

[32] Erdö F, Trapp T, Mies G, Hossmann KA (2004). Immunohistochemical analysis of protein expression after middle cerebral artery occlusion in mice. Acta Neuropathol..

[33] Zhang N (2016). SIRT1 promotes metastasis of human osteosarcoma cells. Oncotarget.

[34] Liu Z (2016). MicroRNA-682-mediated downregulation of PTEN in intestinal epithelial cells ameliorates intestinal ischemia-reperfusion injury. Cell Death & Dis..

[35] Ghatak S (2015). Barrier function of the repaired skin is disrupted following arrest of dicer in keratinocytes. Mol. Ther. J. Am. Soc. Gene Ther..

[36] Ware LB, Matthay MA (2000). The acute respiratory distress syndrome—NEJM. JAMA J. Am. Med. Assoc..

[37] Schwarz B (1999). [Intestinal ischemic reperfusion syndrome: pathophysiology, clinical significance, therapy]. Wien. Klin. Wochenschr..

[38] Stenvang J, Silahtaroglu AN, Lindow M, Elmen J, Kauppinen S (2008). The utility of LNA in microRNA-based cancer diagnostics and therapeutics. Semin. Cancer Biol..

[39] Fluiter K (2003). In vivo tumor growth inhibition and biodistribution studies of locked nucleic acid (LNA) antisense oligonucleotides. Nucleic Acids Res..

[40] Janssen HL (2013). Treatment of HCV infection by targeting microRNA. New Engl. J. Med..

[41] Wen Q (2016). MiR-381-3p regulates the antigen-presenting capability of dendritic cells and represses antituberculosis cellular immune responses by targeting CD1c. J. Immunol..

[42] Xu Z (2017). MicroRNAs upregulated during HIV infection target peroxisome biogenesis factors: Implications for virus biology, disease mechanisms and neuropathology. PLoS. Pathog..

[43] Ban K, Peng Z, Kozar RA (2013). Inhibition of ERK1/2 worsens intestinal ischemia/reperfusion injury. PLoS One.

[44] De Miranda BR (2013). Neuroprotective efficacy and pharmacokinetic behavior of novel anti-inflammatory para-phenyl substituted diindolylmethanes in a mouse model of Parkinson’s disease. J. Pharmacol. Exp. Ther..

[45] Li Y (2017). 6-Gingerol protects intestinal barrier from ischemia/reperfusion-induced damage via inhibition of p38 MAPK to NF-κB signalling. Pharmacol. Res..

[46] Tovah W, Baier SR, Janos Z (2015). The intestinal transport of bovine milk exosomes is mediated by endocytosis in human colon carcinoma Caco-2 cells and rat small intestinal IEC-6 cells. J. Nutr..

[47] Chiu CJ, Mcardle AH, Brown R, Scott HJ, Gurd FN (1970). Intestinal mucosal lesion in low-flow states: I. A morphological, hemodynamic, and metabolic reappraisal. Arch. Surg..

[48] Eckhoff DE, Bilbao G, Frenette L, Thompson JA, Contreras JL (2002). 17-Beta-estradiol protects the liver against warm ischemia/reperfusion injury and is associated with increased serum nitric oxide and decreased tumor necrosis factor-alpha. Surgery.

[49] Mikawa K, Nishina K, Takao Y, Obara H (2003). ONO-1714, a nitric oxide synthase inhibitor, attenuates endotoxin-induced acute lung injury in rabbits. Anesth. Analg..

[50] Chassin C (2012). MicroRNA-146a-mediated downregulation of IRAK1 protects mouse and human small intestine against ischemia/reperfusion injury. EMBO Mol. Med..

