# Supplementary material for: miR-381-3p knockdown improves intestinal epithelial proliferation and barrier function after intestinal ischemia/reperfusion injury by targeting nurr1
Source: Cell Death Dis. 2018 Mar 14;9(3):411. doi: 10.1038/s41419-018-0450-z (PMC5852084; doi:10.1038/s41419-018-0450-z)
Supplement: Supplementary file 1 — Supplementary Figure 1(DOC 83 kb) [file 41419_2018_450_MOESM1_ESM.doc]

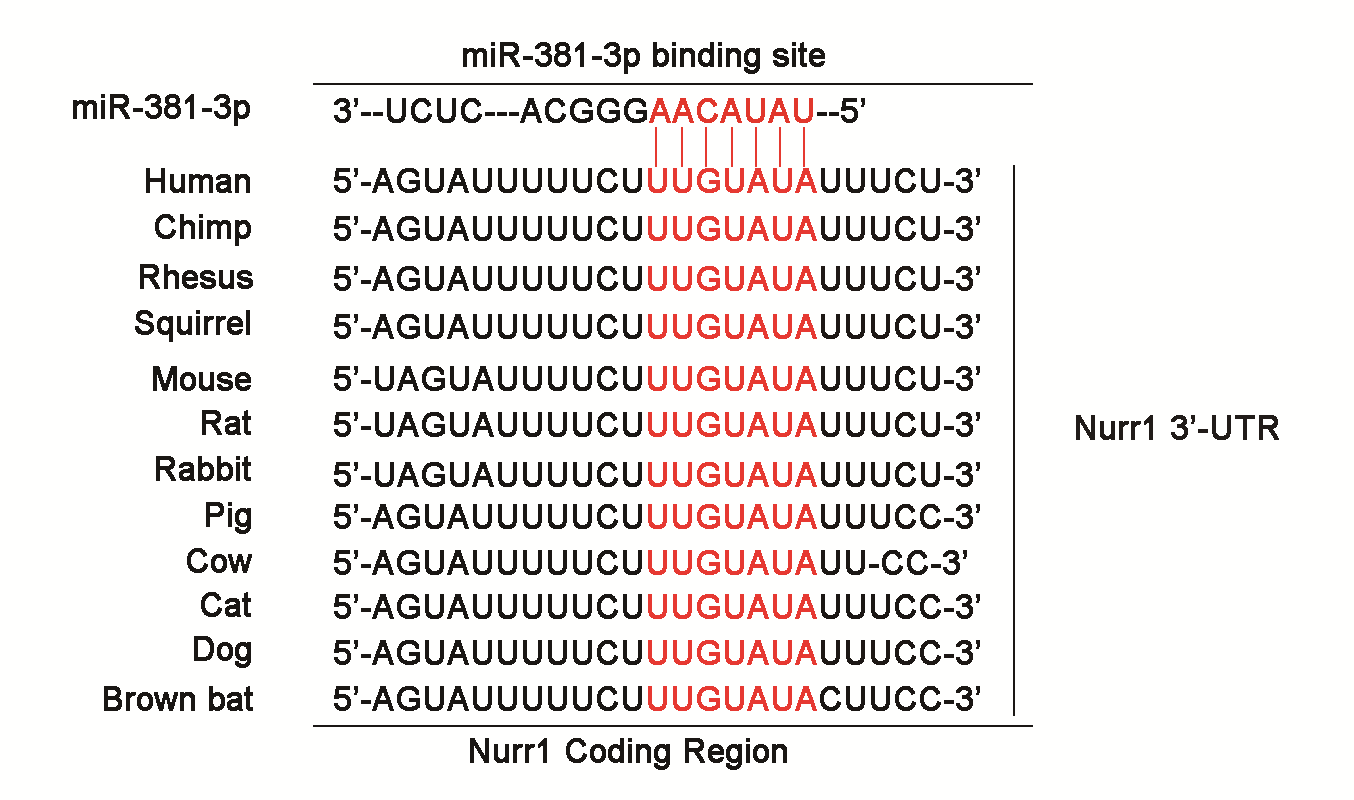
**Supplementary Figure 1. miR-381-3p targets Nurr1 in several organisms.**

A conserved miR-381-3p binding site was identified within the Nurr1 3'-UTR in many organisms in the TargetScan database (http://www.targetscan.org/). The potential complementary residues are shown in red.
