# Supplementary material for: miR-381-3p knockdown improves intestinal epithelial proliferation and barrier function after intestinal ischemia/reperfusion injury by targeting nurr1
Source: Cell Death Dis. 2018 Mar 14;9(3):411. doi: 10.1038/s41419-018-0450-z (PMC5852084; doi:10.1038/s41419-018-0450-z)
Supplement: Supplementary file 2 — Supplementary Figure 2(DOC 264 kb) [file 41419_2018_450_MOESM2_ESM.doc]

**
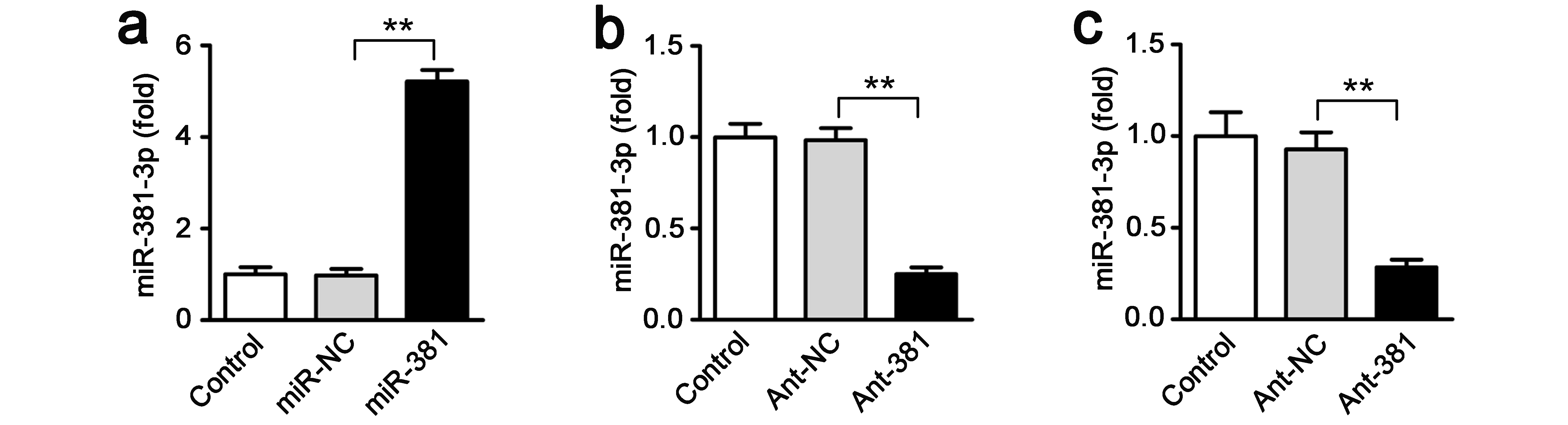
Supplementary Figure 2. Agomir- and antagomir-mediated changes in miR-381-3p.** **(a** and **b)** Caco-2 cells were transfected with miR-381-3p agomir (miR-381), the agomir negative control (miR-NC), miR-381-3p antagomir (ant-381) or the antagomir negative control (ant-NC). qRT-PCR showing miR-381-3p expression, n=6. **(c)** IEC-6 cells were infected with miR-381-3p antagomir (ant-381) and the antagomir negative control (ant-NC), qRT-PCR showing miR-381-3p expression, n=6. **P<0.01. The error bars describe the standard deviation.
