# Supplementary material for: miR-381-3p knockdown improves intestinal epithelial proliferation and barrier function after intestinal ischemia/reperfusion injury by targeting nurr1
Source: Cell Death Dis. 2018 Mar 14;9(3):411. doi: 10.1038/s41419-018-0450-z (PMC5852084; doi:10.1038/s41419-018-0450-z)
Supplement: Supplementary file 3 — Supplementary Table 1(DOC 175 kb) [file 41419_2018_450_MOESM3_ESM.doc]

**Supplementary Table 1**: Differential miRNAs expression in the mouse intestine after I/R injury.

| ID (probes) | miRNA name | FC (injury vs sham) | p-value | Genomic coordinates |
| --- | --- | --- | --- | --- |
| 169344 | mmu-miR-3473b | 11.193394 | 0.034162 | chr10:41670739-41670793［＋］ |
| 27855 | mmu-miR-763 | 9.845511 | 0.007256 | chr10:120447991-120448110［－］ |
| 169291 | mmu-miR-5126 | 9.022414 | 0.026734 | chr1:84695839-84695919［＋］ |
| 168787 | mmu-miR-5114 | 8.292180 | 0.017838 | chr19:44303171-44303231［＋］ |
| 146172 | mmu-miR-1892 | 7.797231 | 0.006758 | chr12:54645933-54646012［－］ |
| 168977 | mmu-miR-5128 | 7.745179 | 0.029920 | chr2:37688201-37688284［－］ |
| 148197 | mmu-miR-3081-5p | 7.707084 | 0.043757 | chr16:44558046-44558129［－］ |
| 42868 | mmu-miR-762 | 7.630351 | 0.015229 | chr7:127708487-127708562［＋］ |
| 11229 | mmu-miR-341-3p | 6.519812 | 0.042879 | chr12:109611500-109611595［＋］ |
| 27574 | mmu-miR-705 | 6.004933 | 0.000292 | chr6:85336292-85336373［－］ |
| 168706 | mmu-miR-5129-5p | 5.984118 | 0.028363 | chr2:45023100-45023177［－］ |
| 148419 | mmu-miR-344c-5p | 5.968830 | 0.001570 | chr7:61837311-61837402［－］ |
| 148037 | mmu-miR-363-5p | 5.807701 | 0.022893 | chrX:52741693-52741767［－］ |
| 42878 | mmu-miR-882 | 5.533136 | 0.027665 | chr12:109682197-109682273［＋］ |
| 42927 | mmu-miR-673-3p | 5.374481 | 0.026764 | chr12:109571990-109572080［＋］ |
| 148446 | mmu-miR-346-3p | 5.373394 | 0.000066 | chr14:34894609-34894706［＋］ |
| 146087 | mmu-miR-1894-3p | 5.335175 | 0.003784 | chr17:35917889-35917969［＋］ |
| 148324 | mmu-miR-1912-5p | 5.324669 | 0.007799 | chrX:147009441-147009527［＋］ |
| 169148 | mmu-miR-5130 | 5.213165 | 0.000257 | chr14:102982549-102982632［－］ |
| 46774 | mcmv-miR-m01-2-5p | 5.151731 | 0.048039 | & |
| 168699 | mmu-miR-5627-5p | 5.124531 | 0.003981 | chr12:44210313-44210373［＋］ |
| 169025 | mmu-miR-5620-5p | 4.849242 | 0.032196 | chr7:7298891-7298946［＋］ |
| 146155 | mmu-miR-2137 | 4.472132 | 0.004839 | chrX:72992079-72992144［＋］ |
| 27568 | mmu-miR-744-5p | 4.259026 | 0.002040 | chr11:65734733-65734832［－］ |
| 146106 | mmu-miR-1931 | 4.118356 | 0.017685 | chr10:93162785-93162903［＋］ |
| 42770 | mmu-miR-665-3p | 3.967244 | 0.012002 | chr12:109586314-109586407［＋］ |
| 146055 | mmu-miR-1954 | 3.945986 | 0.004645 | chr2:32652329-32652414［＋］ |
| 148172 | mmu-miR-216a-3p | 3.695503 | 0.005513 | chr11:28757012-28757083［＋］ |
| 148306 | mmu-miR-381-3p | 3.669091 | 0.046678 | chr12:109726822-109726896［＋］ |
| 17489 | mmu-miR-710 | 3.592656 | 0.045102 | chr8:64514332-64514441［－］ |
| 27575 | mmu-miR-711 | 3.369931 | 0.000815 | chr9:108969922-108970003［＋］ |
| 168751 | mmu-miR-5122 | 3.368119 | 0.024907 | chr4:133369776-133369864［＋］ |
| 148249 | mghv-miR-M1-6-5p | 3.339641 | 0.047084 | U97553: 1319-1381 |
| 146145 | mmu-miR-1895 | 3.252165 | 0.023401 | chr3:134240505-134240583［－］ |
| 42638 | mmu-miR-23a-5p | 3.240833 | 0.013770 | chr8:84208518-84208592［＋］ |
| 148647 | mmu-miR-3470b | 3.185334 | 0.009695 | chr16:44013852-44013977［＋］ |
| 168738 | mmu-miR-5127 | 3.016921 | 0.007698 | chr18:81992010-81992080［－］ |
| 146176 | mmu-miR-1971 | 2.906138 | 0.016559 | chr14:78191373-78191478［－］ |
| 146004 | mmu-miR-2136 | 2.857889 | 0.038310 | chr9:104426113-104426187［＋］ |
| 146082 | mmu-miR-1956 | 2.834246 | 0.001571 | chr3:138526421-138526485［＋］ |
| 148076 | mmu-miR-3103-5p | 2.819921 | 0.000983 | chr7:128288369-128288435［－］ |
| 146029 | mmu-miR-365-2-5p | 2.695785 | 0.015888 | chr11:79726400-79726511［＋］ |
| 17904 | mmu-miR-185-3p | 2.601658 | 0.018015 | chr16:18327401-18327465［－］ |
| 146057 | mmu-miR-1967 | 2.565527 | 0.014175 | chr8:124022641-124022722［＋］ |
| 148200 | mmu-miR-3100-3p | 2.540339 | 0.003540 | chr7:19086828-19086892［＋］ |
| 11235 | mmu-miR-351-5p | 2.472819 | 0.035061 | chrX:53053255-53053353［－］ |
| 148416 | mmu-miR-3102-5p | 2.460961 | 0.014618 | chr7:100882306-100882409［－］ |
| 148146 | mmu-miR-3076-3p | 2.460336 | 0.007436 | chr14:30572149-30572208［＋］ |
| 42702 | mmu-miR-30c-1-3p | 2.371741 | 0.000939 | chr4:120769534-120769622［－］ |
| 11256 | mmu-miR-470-5p | 2.319389 | 0.007955 | chrX:66813951-66814025［－］ |
| 148473 | mmu-miR-3473a | 2.311862 | 0.042604 | chrX:162874918-162874995［－］ |
| 42502 | mmu-miR-204-3p | 2.272732 | 0.037166 | chr19:22750605-22750672［＋］ |
| 17818 | mmu-miR-27a-5p | 2.199209 | 0.033457 | chr8:84208672-84208758［＋］ |
| 148128 | mmu-miR-3090-5p | 2.182288 | 0.008933 | chr2:133564708-133564794［＋］ |
| 42703 | mmu-miR-490-3p | 2.168884 | 0.034355 | chr6:36421742-36421825［＋］ |
| 146133 | mmu-miR-1936 | 2.057370 | 0.028587 | chr12:102684928-102685020［－］ |
| 17273 | mghv-miR-M1-6-3p | 2.033355 | 0.015719 | U97553: 1319-1381［＋］ |
| 17597 | mmu-miR-467b-3p | 0.498741 | 0.007051 | chr2:10481248-10481320［＋］ |
| 145840 | mmu-let-7f-1-3p | 0.496541 | 0.032432 | chr13:48537829-48537917［－］ |
| 46239 | mmu-miR-1191a | 0.491917 | 0.015051 | chr7:27205536-27205655［＋］ |
| 42576 | mmu-miR-342-5p | 0.489004 | 0.021302 | chr12:108658620-108658718［＋］ |
| 11247 | mmu-miR-434-5p | 0.487203 | 0.046601 | chr12:109594506-109594599［＋］ |
| 148166 | mmu-miR-3069-3p | 0.485048 | 0.027664 | chr12:105031077-105031141［－］ |
| 146221 | mmu-miR-669c-5p | 0.483792 | 0.045417 | chr2:10509296-10509404［＋］ |
| 11023 | mmu-miR-222-3p | 0.483059 | 0.010242 | chrX:19146893-19146971［－］ |
| 148586 | mmu-miR-3087-3p | 0.458113 | 0.016949 | chr2:25442779-25442834［＋］ |
| 147701 | mmu-miR-491-3p | 0.449850 | 0.011272 | chr4:88122040-88122125［＋］ |
| 42894 | mmu-miR-466e-5p | 0.448451 | 0.038574 | chr2:10479088-10479171［＋］ |
| 146192 | mmu-miR-669m-3p | 0.443463 | 0.039945 | chr2:10512790-10512887［＋］ |
|  |  |  |  | chr2:10513434-10513531［＋］ |
| 11227 | mmu-miR-329-3p | 0.442657 | 0.004113 | chr12:109713481-109713577［＋］ |
| 169053 | mmu-miR-130b-5p | 0.440740 | 0.016561 | chr16:17124061-17124142［－］ |
| 148426 | mmu-miR-466a-3p | 0.440733 | 0.016378 | chr2:10507918-10507990［＋］ |
| 148426 | /mmu-miR-466b-3p | 0.440733 | 0.016378 | chr2:10474219-10474300［＋］ |
|  |  |  |  | chr2:10498685-10498766［＋］ |
|  |  |  |  | chr2:10503565-10503645［＋］ |
|  |  |  |  | chr2:10486423-10486512［＋］ |
|  |  |  |  | chr2:10488887-10488974［＋］ |
|  |  |  |  | chr2:10493798-10493887［＋］ |
|  |  |  |  | chr2:10496268-10496355［＋］ |
|  |  |  |  | chr2:10476628-10476713［＋］ |
| 148426 | / mmu-miR-466e-3p | 0.440733 | 0.016378 | chr2:10479088-10479171［＋］ |
| 148426 | /mmu-miR-466p-3p | 0.440733 | 0.016378 | chr2:10479088-10479171［＋］ |
| 46917 | mmu-miR-205-5p | 0.420685 | 0.007118 | chr1:193507463-193507530［－］ |
| 46346 | mmu-miR-669e-5p | 0.410410 | 0.029215 | chr2:10467495-10467613［＋］ |
| 146199 | mmu-miR-1961 | 0.409306 | 0.031222 | chr5:92788451-92788562［－］ |
| 145822 | mmu-miR-214-5p | 0.407446 | 0.001671 | chr1:162223368-162223477［＋］ |
| 29852 | mmu-miR-9-3p | 0.400451 | 0.043824 | chr3:88215598-88215686［＋］ |
|  |  |  |  | chr13:83738814-83738885［＋］ |
|  |  |  |  | chr7:79505264-79505353［＋］ |
| 148052 | mmu-miR-374c-3p | 0.392697 | 0.001800 | chrX:103573085-103573133［＋］ |
| 145643 | mmu-miR-382-5p | 0.391265 | 0.006729 | chr12:109733771-109733846［＋］ |
| 30768 | mmu-miR-674-5p | 0.389131 | 0.024034 | chr2:117185127-117185226［＋］ |
| 145857 | mmu-miR-154-5p | 0.387470 | 0.028720 | chr12:109738433-109738498［＋］ |
| 17851 | mmu-miR-200c-5p | 0.380702 | 0.003265 | chr6:124718322-124718390［－］ |
| 17304 | mmu-miR-683 | 0.377058 | 0.026841 | chr13:50544626-50544734［－］ |
|  |  |  |  | chr13:50600972-50601080［－］ |
| 11184 | mmu-miR-99b-5p | 0.372188 | 0.000741 | chr17:17830188-17830257［＋］ |
| 145757 | mmu-miR-33-3p | 0.369506 | 0.023012 | chr15:82198122-82198190［＋］ |
| 31867 | mmu-miR-145a-3p | 0.366378 | 0.017677 | chr18:61647825-61647894［－］ |
| 42551 | mmu-miR-122-3p | 0.364302 | 0.034773 | chr18:65248861-65248926［＋］ |
| 148259 | mmu-miR-3070-5p | 0.363458 | 0.011711 | chr12:109587943-109588031［＋］ |
|  |  |  |  | chr12:109588592-109588680［＋］ |
| 148175 | mmu-miR-1843a-3p | 0.362445 | 0.002329 | chr12:80391613-80391677［－］ |
| 29190 | mmu-miR-708-5p | 0.353596 | 0.004295 | chr7:96249424-96249532［＋］ |
| 11260 | mmu-miR-151-5p | 0.349841 | 0.047731 | chr15:73254815-73254882［－］ |
| 29490 | mmu-miR-7a-5p | 0.347490 | 0.004813 | chr13:58392779-58392886［－］ |
|  |  |  |  | chr7:78888277-78888373［＋］ |
| 145637 | mmu-miR-187-3p | 0.342912 | 0.000910 | chr18:24429110-24429170［－］ |
| 28161 | mmu-miR-380-5p | 0.334955 | 0.001265 | chr12:109711803-109711863［＋］ |
| 30831 | mmu-miR-804 | 0.327139 | 0.001938 | chr11:50357785-50357879［－］ |
| 169408 | mmu-miR-181d-5p | 0.323328 | 0.001461 | chr8:84178716-84178787［－］ |
| 168966 | mmu-miR-28a-5p | 0.318382 | 0.007355 | chr16:24827855-24827940［＋］ |
| 168966 | /mmu-miR-28c | 0.318382 | 0.007355 | chr15:53614205-53614279［－］ |
| 10985 | mmu-miR-191-5p | 0.317612 | 0.005689 | chr9:108568319-108568392［＋］ |
| 14285 | mmu-miR-487b-3p | 0.305827 | 0.022724 | chr12:109727333-109727414［＋］ |
| 30442 | mmu-miR-802-5p | 0.303543 | 0.019110 | chr16:93369720-93369816［＋］ |
| 145638 | mmu-miR-29a-5p | 0.295315 | 0.011284 | chr6:31062660-31062747［－］ |
| 148098 | mmu-miR-374b-5p | 0.293246 | 0.000499 | chrX:103573060-103573154［－］ |
| 148278 | mmu-miR-138-2-3p | 0.291908 | 0.012347 | chr8:94324311-94324381［＋］ |
| 42739 | mmu-miR-339-5p | 0.289142 | 0.000133 | chr5:139369650-139369745［－］ |
| 148220 | mmu-miR-3093-5p | 0.288362 | 0.037610 | chr3:88215171-88215257［＋］ |
| 42978 | mmu-miR-466a-3p | 0.285213 | 0.015387 | chr2:10507918-10507990［＋］ |
| 42978 | /mmu-miR-466e-3p | 0.285213 | 0.015387 | chr2:10479088-10479171［＋］ |
| 148589 | mmu-miR-3109-5p | 0.277083 | 0.048823 | chr9:69456944-69457031［＋］ |
| 42736 | mmu-miR-148b-5p | 0.270336 | 0.001565 | chr15:103285125-103285221［＋］ |
| 148190 | mmu-miR-3091-3p | 0.255658 | 0.049393 | chr2:180257536-180257611［＋］ |
| 11077 | mmu-miR-363-3p | 0.252208 | 0.032769 | chrX:52741693-52741767［－］ |
| 14301 | mmu-miR-361-5p | 0.244289 | 0.000017 | chrX:113074824-113074893［－］ |
| 148548 | mmu-miR-3090-3p | 0.243280 | 0.019876 | chr2:133564708-133564794［＋］ |
| 42887 | mmu-miR-331-3p | 0.242996 | 0.010097 | chr10:93963768-93963863［－］ |
| 42619 | mmu-miR-709 | 0.242386 | 0.021056 | chr8:84086099-84086186［＋］ |
| 148668 | mmu-miR-378a-3p | 0.234157 | 0.000637 | chr18:61397835-61397900［－］ |
| 148531 | mmu-miR-544-5p | 0.233843 | 0.000699 | chr12:109729325-109729402［＋］ |
| 19582 | mmu-miR-106b-5p | 0.229099 | 0.000315 | chr5:138165737-138165818［－］ |
| 11246 | mmu-miR-434-3p | 0.208481 | 0.042601 | chr12:109594506-109594599［＋］ |
| 148484 | mmu-miR-3084-3p | 0.208162 | 0.000019 | chr19:24942235-24942303［－］ |
| 17872 | mmu-miR-148a-5p | 0.204363 | 0.007578 | chr6:51269812-51269910［－］ |
| 168937 | mmu-miR-138-1-3p | 0.193043 | 0.015484 | chr9:122682876-122682974［＋］ |
| 147536 | mmu-miR-107-5p | 0.187422 | 0.037946 | chr19:34820687-34820773［－］ |
| 46979 | mmu-miR-669h-3p | 0.185440 | 0.041027 | chr2:10518155-10518279［＋］ |
| 145701 | mmu-miR-668-3p | 0.174757 | 0.006020 | chr12:109734732-109734797［＋］ |
| 148610 | mmu-miR-26a-1-3p | 0.163711 | 0.017315 | chr9:119031796-119031885［＋］ |
| 17669 | mmu-miR-690 | 0.160442 | 0.007431 | chr16:28599935-28600043［－］ |
| 146163 | mmu-miR-224-3p | 0.157305 | 0.047190 | chrX:72261031-72261112［－］ |
| 148035 | mmu-miR-3084-5p | 0.115103 | 0.013914 | chr19:24942235-24942303［－］ |
|  |  |  |  | chr19:60774329-60774397［－］ |
| 147283 | mmu-miR-137-5p | 0.104963 | 0.048739 | chr3:118433857-118433929［＋］ |
| 148428 | mmu-miR-3069-5p | 0.067800 | 0.047113 | chr12:105031077-105031141［－］ |
| 46217 | mcmv-miR-m108-1-3p | 0.066992 | 0.013158 | & |

The microarray analysis identified 131 differentially expressed miRNAs in the I/R group, all of which exhibited a p-value<0.05 calculated by t-test compared with the sham group (n=3 per group). Each miRNA has a unique probe ID (Exiqon, Vedbaek, Denmark), but some miRNAs may have two different probes. Some homologous miRNAs cannot be distinguished by the probe. FC, fold change.

&: only experimental evidence. Not clear in the mouse genome.

All genomic coordinates information is derived from the miRBase database (http://www.mirbase.org). Some miRNAs may have several different genomic coordinates, which results in many spaces in the table.
